# Biodegradable PVA–Alginate Bio-Based Polymers Incorporating Cardanol-Based Polyols for Antibacterial Applications

**DOI:** 10.3390/polym17202792

**Published:** 2025-10-18

**Authors:** Da Hae Lee, Hee Ju Ahn, Jaekyoung Lee, Hee Chul Woo

**Affiliations:** Department of Chemical Engineering, Pukyong National University, 45 Yongso-ro, Nam-gu, Busan 48513, Republic of Korea; ekgo2364@pukyong.ac.kr (D.H.L.);

**Keywords:** bio-based polymer, cardanol, polyvinyl alcohol, alginate, biomass, crosslinking

## Abstract

The extensive use of petroleum-based plastics has caused serious environmental concerns; thus, the need for biodegradable alternatives is essential. Here, we present eco-friendly bio-based polymers prepared by crosslinking poly(vinyl alcohol) (PVA) and alginate (ALG) with glutaraldehyde, while incorporating cardanol-derived polyols (PCD) to add antibacterial functionality. The synthesized bio-based polymers were characterized by FT-IR, XRD, and TGA. FT-IR confirmed sufficient crosslinking between PVA and ALG, whereas XRD revealed a minor decrease in crystallinity. Thermogravimetric analysis showed enhanced thermal stability with increasing ALG contents, as the residual mass increased from 8 wt% (PVA only) to 19–31% (PVA:ALG = 80:20–60:40). Swelling behavior was strongly governed by ALG, with higher ratios promoting water uptake up to 130%, whereas PCD reduced swelling due to increased hydrophobicity. Antibacterial assays indicated complete inactivation of *Escherichia coli* and *Staphylococcus aureus* within 10–60 min depending on the polymer composition. These results demonstrate that tuning the PVA:ALG ratio and PCD content allows precise control of physicochemical properties. Overall, the developed PVA–ALG/PCD bio-based polymers represent a versatile and sustainable platform for eco-friendly packaging, biomedical, and water treatment applications.

## 1. Introduction

In modern industrial society, petroleum-derived plastics have been extensively utilized in packaging, films, containers, and a variety of consumer products due to their low cost, good durability, heat resistance, and lightweight properties. However, their increasing consumption has resulted in an unprecedented accumulation of plastic waste, which poses a severe environmental challenge. From 1950 to 2015, the global production of plastics reached approximately 8.3 billion tons, with about 60% being discarded, landfilled, or released into the environment, while the recycling rate remained as low as 7.2% [1]. Petroleum-based plastics are non-biodegradable, requiring several decades to centuries for natural decomposition. Currently, plastics are mainly disposed of via incineration and landfilling, which have significant drawbacks. Incineration produces greenhouse gases, such as carbon dioxide and toxic gases, as by-products, while landfilling or marine disposal cause long-term environmental issues in ecosystems, leading to ecological imbalance. Consequently, the global community has strengthened regulatory measures on single-use plastics, with more than 150 countries implementing legislation to restrict consumption [2,3,4]. In parallel, researchers have been devoted to developing biodegradable plastics, such as bio-based polymers, as sustainable alternatives.

Biodegradable plastics are defined as polymers that can be decomposed into carbon dioxide, water, and biomass by microorganisms under specific environmental conditions. They can be categorized into petroleum-based biodegradable polymers, such as polyvinyl alcohol (PVA), polybutylene succinate (PBS), and polybutylene adipate terephthalate (PBAT), and bio-based biodegradable polymers, including polylactic acid (PLA), thermoplastic starch (TPS), and polyhydroxyalkanoates (PHA). Bio-based polymers, derived from renewable resources such as starch, cellulose, and chitin, have gained particular interest due to their low toxicity, biocompatibility, and reduced carbon footprint, aligning well with carbon neutrality. Nevertheless, despite their environmental advantages, many bio-based polymers still suffer from mechanical weaknesses, low stability, and limited processability, which limit their substitution of conventional plastics in industrial applications [5,6]. Among them, alginate (ALG), a naturally derived polysaccharide obtained primarily from brown seaweed, is one of the most promising candidates for bio-based polymers [7,8,9,10]. Alginate exhibits inherent biocompatibility, non-toxicity, high hydrophilicity, and excellent water absorption capacity, which have facilitated its application in wound dressings, drug delivery carriers, and food additives [8,9]. However, like other bio-based materials, alginate still exhibits low mechanical strength and poor thermal stability, which restricts its direct application as structural material or packaging film [10]. Therefore, strategies to enhance physicochemical properties of alginate-based polymers are critical for extending their practical applicability.

To overcome these limitations, blending strategies that combine the advantageous properties of different polymers have been extensively investigated. Poly(vinyl alcohol) (PVA) is particularly attractive owing to its water-solubility, non-ionic character, film-forming ability, mechanical robustness, hydrophilicity, and biodegradability [11,12,13,14,15,16,17]. Blending alginate with PVA provides a rational approach as PVA can form extensive hydrogen bonding with the hydroxyl and carboxyl groups of alginates, thereby improving intermolecular interactions and mechanical integrity. In addition, the good film-forming ability and mechanical strength of PVA would be beneficial for enhancing mechanical strength and processability of alginate-based materials. The complementary characteristics of PVA’s mechanical robustness and ALG’s excellent biocompatibility enable the formation of composite hydrogels or films with enhanced physical and biological properties. Several studies have reported that PVA–alginate blends exhibit improved swelling capacity, elasticity, and thermal stability, as well as favorable biocompatibility, making them suitable for biomedical applications, such as wound dressings and tissue engineering scaffolds [16,18,19,20]. Nonetheless, the lack of sufficient antimicrobial activity of PVA–ALG composites remains a major limitation that restricts their broader utilization in healthcare and packaging industries.

In this context, the incorporation of antimicrobial agents into biodegradable polymer matrices has emerged as a promising strategy. For example, alginate-based films incorporated with ZnO nanoparticles and citronella essential oil showed enhanced antimicrobial activity, highlighting the potential of combining bio-based polymers with functional additives [21]. Among the various candidates, cardanol—a phenolic lipid derived from cashew nut shell liquid (CNSL), an abundant agricultural by-product—has attracted increasing attention. Cardanol contains a long aliphatic side chain with unsaturated bonds, exhibiting unique physicochemical properties. Moreover, cardanol exhibits intrinsic antimicrobial, antioxidant, and anticorrosive properties, attributed to its phenol group, which make it highly suitable as a functional additive in bio-based polymers [22,23]. Recent studies have demonstrated that cardanol-derived polyols, when introduced into polyurethane and other polymer systems, not only improve the tensile strength and thermal stability but also impart significant antibacterial activity against pathogens such as *Escherichia coli* and *Staphylococcus* aureus [23,24,25,26,27]. These findings strongly suggest that cardanol is an effective bio-based additive for enhancing both the performance and functionality of biodegradable polymers [25,26,27,28,29]. However, the effect of cardanol on the structural, mechanical, and functional properties of PVA–ALG-based bio-based polymers has not been systematically investigated. In addition, the antimicrobial performance of crosslinked PVA–ALG–PCD-based bio-based polymers remains to be elucidated.

In this work, we developed and characterized novel bio-based polymers composed of alginate (ALG) and PVA, with antimicrobial properties introduced via polyols derived from cardanol (PCD) via crosslinking among raw materials. The structural, physicochemical, and antibacterial properties of the prepared PVA–alginate–cardanol composites were systematically evaluated, with the goal of elucidating their potential as eco-friendly materials for practical applications. By combining renewable biomass resources with functional bio-additives, this work contributes to advancing biodegradable polymer technology and addressing the urgent need for sustainable alternatives to petroleum-based plastics.

## 2. Materials and Methods

### 2.1. Materials

Cardanol (CD) was provided by Chemifolio Co., Ltd. (Sinseon-Ro, Republic of Korea) Poly(vinyl) alcohol (99%, MW = 89,000–98,000) and glutaraldehyde (50 wt% solution) were purchased from Sigma-Aldrich. Sodium Alginate (80–120 cP) was purchased from Wako Chemicals (Doshomachi, Republic of Korea). Hydrochloric acid (35.0–37.0%) was purchased from Samchun Chemicals (Gyeonggi-do, Republic of Korea).

### 2.2. Preparation

#### 2.2.1. Synthesis of Polyols Derived from Cardanol

Epoxidized cardanol (ECD), a precursor for PCD, was synthesized from cardanol following the procedure described in our previous work [30]. For the synthesis of polyols derived from cardanol (PCD), epoxidized cardanol (200 g) was reacted with 11% sulfuric acid solution (200 mL) at 70 °C for 5 h, followed by cooling to room temperature. After phase separation, the organic layer was extracted with ethyl acetate (99.5%, 200 mL) and stirred. The mixture was subsequently neutralized, dried, and distilled using the same procedure, yielding dark brown products.

#### 2.2.2. Fabrication of Bio-Based Polymers

Bio-based polymer was synthesized in 100 mL round bottom flask equipped with a stirrer, reflux cooler, and thermometer. Hydrochloric acid solution (pH 2) and PVA were stirred at 95 °C, 1 h. Sodium alginate was added and stirred at 95 °C, 1 h. The concentration of mixture was 10 wt% (PVA: ALG weight ratio ranged from 20:80 to 40:60). After mixing, the liquid was cooled at 60 °C and polyols derived from cardanol (PCD) dissolved in ethanol were added. The amounts of PCD were added 0–2 wt% with respect to total weight of PVA and ALG. After 30 min stirring, the crosslinker, glutaraldehyde (GLU), was added to 10–20 wt% for total weight of PVA and ALG. The mixture was stirred for 30 min. All sample compositions were chosen based on extension of our previous studies [30,31], and Table S1 summarizes the samples studied in this work.

The prepared bio-based polymers were fabricated in two forms as films and gels. The films were cast on PET film using 250 μm film applicator (YBA-6, YOSHIMITSU (Kyoto, Japan)) and gels were cast in Petri dish. All bio-based polymer samples were dried at 30 °C overnight and named as PVA(wt%)–ALG(wt%)/PCD(wt%)/GLU(wt%). We used the prepared bio-based polymers in films for characterization of FT-IR, XRD, TGA, and antibacterial test and gels for the swelling test.

### 2.3. Characterization

#### 2.3.1. FT-IR

Fourier Transform-Infrared Spectroscopy (FT-IR) was employed to identify specific chemical groups. Spectra were recorded using an FT-IR spectrometer (FT-4100, JASCO International Co., Ltd., Tokyo Japan) in attenuated total reflection (ATR) mode over a wavenumber range of 4000–600 cm^−1^, with a resolution of 4 cm^−1^ and a mirror speed of 2 mm/s.

#### 2.3.2. XRD

X-ray diffraction (XRD) was employed to evaluate the crystallinity index (*CI*). Measurements were carried out using an X’Pert3-Powder diffractometer (Malvern PANalytical) equipped with Cu-Kα radiation (λ = 1.54 Å), operating at 40 eV and 30 mA. Data were collected over a 2θ range of 10–80° at a scanning rate of 2°/min. The CI was calculated according to the method reported by Abral et al. [32]. Specifically, the maximum intensity (I_T_) at the main peak, corresponding to the (101) lattice plane at 2θ = 19.5°, and the minimum intensity (I_A_) prior to the growth of the main peak were determined. The *CI* was then obtained using the following Equation (1):

(1) $$CI\;\left(\%\right)=\{\left(I_{T}-I_{A}\right)∕I_{T}\}\times 100$$

#### 2.3.3. TGA

Thermogravimetric analysis (TGA) was conducted to evaluate the thermal properties of the polymers. Measurements were performed using a thermoanalyzer (TGA/DSC1, Mettler-Toledo AG). Samples were heated from 25 °C to 600 °C at a rate of 10 °C/min under a nitrogen flow of 50 mL/min.

#### 2.3.4. Swelling Test

The swelling behavior of the hydrogels was evaluated according to the JIS K 7223 method (Japanese Industrial Standard) [33]. Samples were weighed and placed in nylon tea bags. Initially, the tea bags were immersed in 0.5 M CaCl_2_ solution for 24 h to prevent uncrosslinked ALG from dissolving in PBS, followed by drying at 40 °C for 48 h. The samples were then re-immersed in phosphate-buffered saline (PBS) at 25 °C for 24 h. After immersion, the tea bags were removed, and excess water was drained for 15 min. The swollen hydrogels were weighed (*W_s_*) and subsequently dried at 40 °C for 48 h to obtain the dry weight (*W_d_*). The swelling ratio was calculated using the following Equation (2):

(2) $$Swelling\;ratio\;\left(\%\right)=\{\left(W_{s}-W_{d}\right)∕W_{d}\}\times 100$$

#### 2.3.5. Antibacterial Test

The time-kill procedure was based on the KS standard (KS M ISO 22196). The bacterial strains used were *Staphylococcus aureus* (*S. aureus*; ATCC 6538P) and *Escherichia coli* (*E. coli*; ATCC 8739). The number of bacteria was measured according to the contact time (1 to 60 min), and the kill rate was calculated. The kill rate was calculated by setting the number of bacteria in the test sample after the contact time as *S_t_*, and the number of bacteria in the control sample after the contact time as *C_t_* with the below Equation (3):

(3) $$Kill\;rate\;\left(\%\right)=\{1-(S_{t}/C_{t})\}\times 100$$

## 3. Results and Discussions

In this work, we prepared a series of bio-based polymers by blending alginate (ALG) derived from brown algae biomass with poly(vinyl alcohol) (PVA). Two raw materials have different physicochemical properties; adjusting the content ratio would affect the physicochemical properties of PVA–ALG bio-based polymers. The content ratio varied from PVA main (PVA–ALG: 80–20 wt%) to ALG main (PVA–ALG: 20–80 wt%). To crosslink each raw material, glutaraldehyde (GLU) was added, and the crosslinking and related changes in physicochemical properties of bio-based polymers are to be characterized by FT-IR, TGA, XRD, and the swelling tests. To impart antimicrobial activity, 1 and 2 wt% of polyols derived from the phenolic lipid cardanol (PCD) were incorporated. To understand how additive PCD affects the properties, PVA–ALG without PCD and with PCD was compared on each characterization analysis. The antimicrobial activity was tested via antibacterial tests.

### 3.1. FT-IR of Bio-Based Polymers

**Figure 1:**
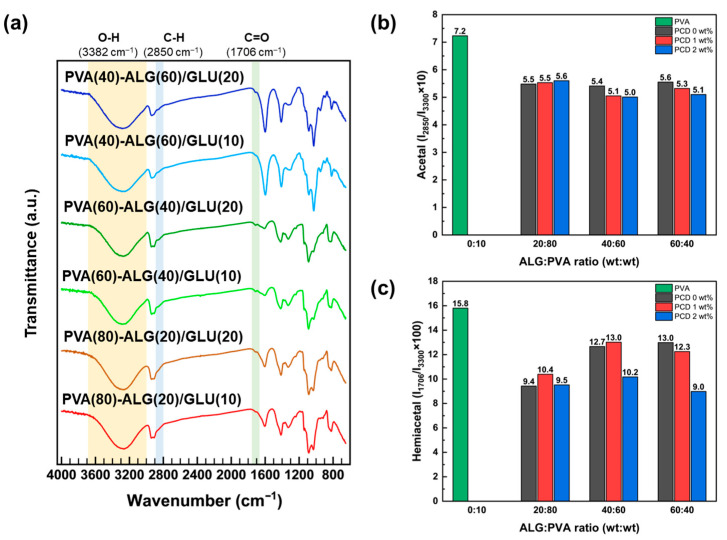
(**a**) FT-IR spectra of PVA–ALG bio-based polymers, ratio of (**b**) acetal (I_2850_/I_3300_) and (**c**) hemiacetal (I_1706_/I_3300_) associated with ALG:PVA ratio and PCD contents.

The chemical structure of the prepared bio-based polymers was characterized by Fourier Transform-Infrared Spectroscopy (FT-IR) to examine the changes in functional groups induced by crosslinking, and the results are presented in Figure 1 and Figures S1 and S2. The characteristic absorption bands and corresponding functional groups are summarized in Tables S2 and S3.

The major raw materials, namely PVA, ALG, and PCD, were characterized and the results are shown in Figure S1. PVA showed a broad absorption band in the range of 3200–3500 cm^−1^, which was attributed to the abundant hydroxyl groups and intermolecular hydrogen bonding between polymer chains. The peak around 2900 cm^−1^ was assigned to C-H stretching vibrations of -CH_2_- groups, while those at 1140 and 1090 cm^−1^ corresponded to C-O stretching, confirming the alcohol structure of PVA [32,34,35,36].

In the case of ALG, a broad band near 3400 cm^−1^ was assigned to O-H stretching, reflecting the abundance of hydroxyl groups in the polysaccharide backbone. Strong peaks at ~1590 and ~1415 cm^−1^ corresponded to the asymmetric and symmetric stretching of -COO^−^ groups, indicating the anionic nature of alginate. Furthermore, peaks in the 1020–1100 cm^−1^ range were attributed to C-O-C and C-O vibrations, which are characteristic of the saccharide structure of alginate and associated with its pyranose ring, a six-membered ring structure composed of five carbon atoms and one oxygen atom [37,38,39,40].

For PCD, the characteristic peaks originating from the precursor cardanol were maintained (C=C stretching vibrations of the aromatic ring at 1589, 1487, and 1456 cm^−1^, C–H stretching vibrations of the alkyl chain at 2925 and 2853 cm^−1^, C-H bending vibration in aromatic ring at 911 and 884 cm^−1^, and the C–O stretching vibration of the phenolic group at 1263 cm^−1^). A broad O–H stretching band appears around 3339 cm^−1^ upon conversion to polyols, indicating the formation of hydroxyl groups through hydrogen bonding [41,42,43,44].

**Scheme 1:**
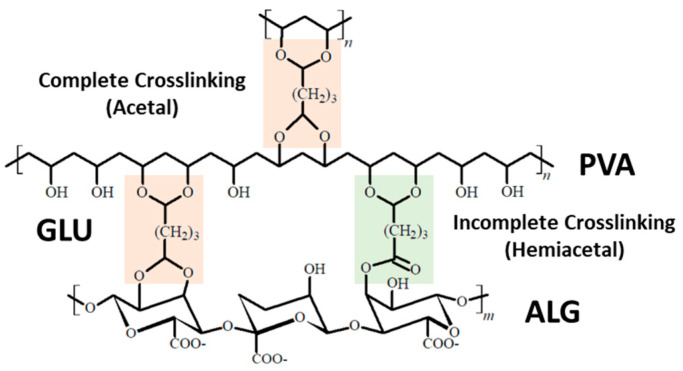
Chemical crosslinking of poly(vinyl) alcohol (PVA), alginate (ALG), and glutaraldehyde (GLU) in the form of complete crosslinking (orange) and incomplete crosslinking (green).

Figure 1a presents the FT-IR spectra of PVA–ALG bio-based polymers with varying ALG contents (20, 40, and 60 wt%) under different contents of the glutaraldehyde (GLU) (10 and 20 wt%). Hydroxyl absorption peaks were observed in the range of 3259–3280 cm^−1^, which shifted to higher wavenumbers with increasing ALG content. This peak shift indicates the formation of diverse hydrogen bonding interactions resulting from ALG incorporation and crosslinking. Peaks observed in the region of 2910–2940 cm^−1^ correspond to C–H stretching vibrations of crosslinked acetal group. Upon the addition of GLU, the crosslinking agent, functional groups generated during complete or incomplete crosslinking were detected, as illustrated in Scheme 1. Previous studies have reported that the C–H stretching vibration of acetal groups appears near 2860 cm^−1^ [45]. Furthermore, the peak at ~1706 cm^−1^ was attributed to the carbonyl group of hemiacetal structures formed after crosslinking, and these two bands can be employed as parameters of the crosslinking degree [12]. Although characteristic peaks associated with ether bridges were also observed, they were difficult to be used due to the interference by abundant oxygen atoms in ALG.

In summary, the crosslinking effect of ALG in PVA–ALG bio-based polymers was evaluated by analyzing the intensity ratios I_2850_/I_3300_ (acetal) and I_1706_/I_3300_ (hemiacetal), and the results are presented in Figure 1b,c.

Figure 1b shows the ratio of C–H stretching vibration (I_2850_) to hydroxyl stretching vibration (I_3300_) during crosslinking. A series of PVA–PCD bio-based polymers exhibited similar I_2850_/I_3300_ values in the range of 5.0–5.6. Compared to pure PVA, this ratio value decreased significantly, which originated from the steric hindrance induced by the addition of ALG, composed of hexose units, as well as the insufficient reactive groups (hydroxyl and carboxyl groups) required for crosslinking. Furthermore, since the I_2850_/I_3300_ values were similar regardless of the ALG content, the content of ALG did not influence the degree of crosslinking significantly [46].

Figure 1c presents the relative formation of acetal groups, expressed as the ratio I_1706_/I_3300_. This ratio value was slightly reduced in PVA–ALG bio-based polymers compared to pure PVA. For 20 wt% ALG content, a greater decrease was observed, which can be explained by the poor miscibility between the relatively low molecular weight PVA (~100,000 Da) matrix and the higher molecular weight ALG (~800,000 Da) when incorporated. Small amounts of ALG might be isolated, leading to localized crosslinking within PVA rather than forming an integrated network. In contrast, at 40 and 60 wt% ALG content, PVA and ALG were more evenly mixed, resulting in a more homogeneous crosslinked bio-based polymers.

**Scheme 2:**
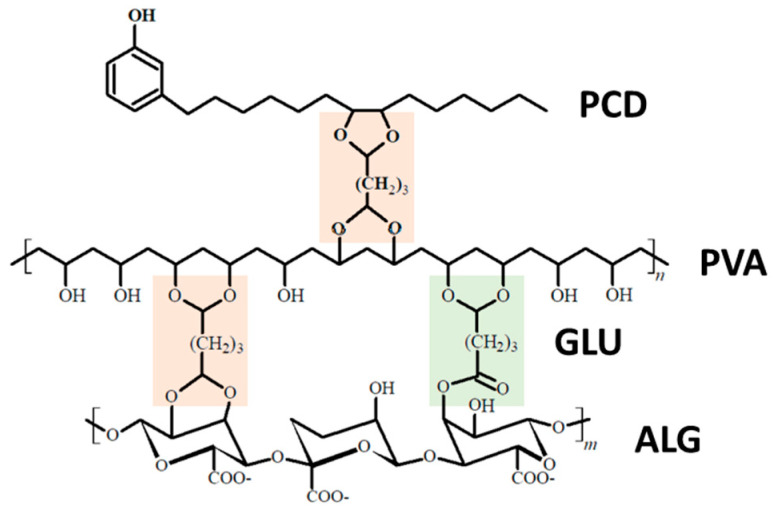
Chemical crosslinking of poly(vinyl) alcohol (PVA), alginate (ALG), polyols derived from cardanol (PCD), and glutaraldehyde (GLU). Orange denotes complete crosslinking and green denotes incomplete crosslinking.

The FT-IR spectra of PVA–ALG bio-based polymers incorporated with PCD are presented in Figure S2, the degree of crosslinking is shown in Figure 1b,c, and the crosslinked PVA–ALG/PCD bio-based polymers are illustrated in Scheme 2.

Figure 1b shows that under 1 and 2 wt% PCD, the PVA–ALG/PCD bio-based polymers exhibited similar I_2850_/I_3300_ values (5.0–5.6), indicating that the addition of small amounts of PCD had minimal influence on the overall crosslinking degree. However, the extent of hemiacetal group formation (Figure 1c) was lower at 2 wt% than at 1 wt%, which suggests that the increased presence of PCD interfered with the penetration and dispersion of GLU, thereby partially hindering the crosslinking reaction.

This result is consistent with the XRD results (Figure S3) to show that the crystallinity index of PVA(60)–ALG(40) bio-based polymers decreased at 1 wt% PCD, but increased at 2 wt% PCD relative to PVA–ALG bio-based polymers without PCD. Increased crystallinity observed at 2 wt% PCD suggests that the extent of disordered crosslinking within the bio-based polymer matrix was reduced [47,48].

Overall, FT-IR analysis showed that the PVA–ALG bio-based polymer exhibited good miscibility within a certain compositional range and underwent effective crosslinking induced by the GLU crosslinker. In addition, the incorporation of a small amount of PCD in PVA–ALG bio-based polymers was confirmed, which would introduce antibacterial properties.

### 3.2. TGA

**Figure 2:**
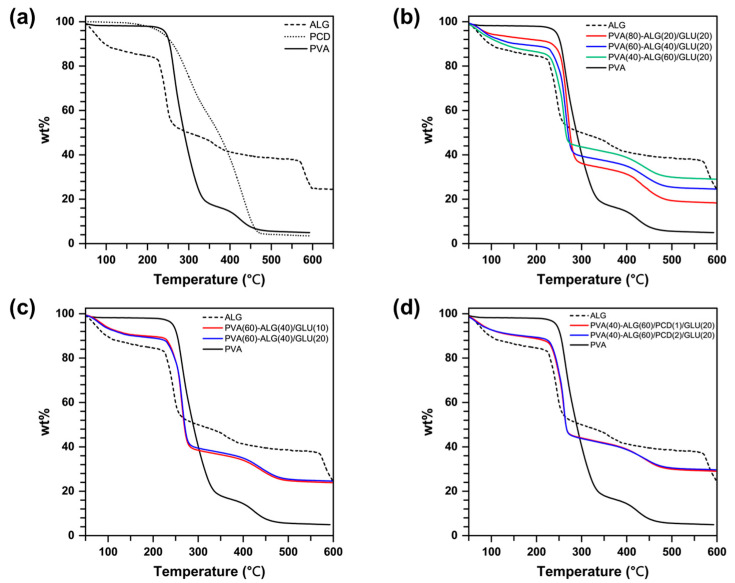
TGA curves for (**a**) base materials, (**b**) PVA–ALG/GLU(20), (**c**) PVA(60)–ALG(40), (**d**) PVA(40)–ALG(60)/PCD/GLU(20) bio-based polymers.

To investigate the thermal degradation behavior of the three major raw materials used for preparing the composite bio-based polymer—PVA, ALG, and PCD—thermogravimetric analysis (TGA) was performed, and the results are presented in Figure 2a.

For PVA, three distinct weight loss stages were observed. In the first stage (25–233 °C), approximately 3.3% weight loss occurred due to the evaporation of adsorbed moisture. The second stage (240–385 °C) corresponded to dehydration of the PVA main chain, chain scission, and degradation of side chains, leading to about 81% weight loss. In the third stage, up to 474 °C, an additional 9.5% decrease was observed because of the further degradation of polyene structures. Above 500 °C, only about 5% of carbonaceous residue remained. These results are consistent with the typical thermal decomposition behavior of PVA reported in previous studies [49,50,51].

For ALG, weight loss in the range of 25–204 °C occurred because of the evaporation of moisture and volatile compounds. The sharp reduction between 218 and 264 °C was associated with the cleavage of glycosidic linkages in the polymeric network. Beyond 264 °C, the weight decreased gradually because the carbonaceous residues were formed along with the retention of Na_2_CO_3_ derived from sodium ions inherently present in alginate. This residue remained in the form of char [52].

For PCD, an initial weight loss of ~2% was observed between 25 and 200 °C due to the evaporation of moisture. Between 200 and 470 °C, decomposition of the cardanol alkyl chains resulted in approximately 89% weight loss, with an inflection point at 351 °C. Up to this temperature, both chain scission of the alkyl groups and oligomer formation through the polymerization of double bonds likely occurred. Subsequently, volatile monomeric species were eliminated up to 470 °C, leaving ~3.5% of carbonaceous residue at 590 °C [53].

To evaluate how the crosslinking among PVA, ALG, and PCD affects the thermal stabilities, systematic TGA was performed on a series of PVA–ALG–PCD bio-based polymers. TGA results are shown in Figure 2b–d. Figure 2b presents TGA results of PVA–ALG bio-based polymers prepared by fixing the GLU crosslinker content at 20 wt% while varying the PVA:ALG ratios to 80:20, 60:40, and 40:60. The thermal decomposition behavior can be divided into four distinct regions. In the low temperature range of 25–220 °C, weight loss increased with higher alginate content, reflecting the inherent characteristics of alginate. In the range of 220–290 °C, a rapid weight loss of 40–52 wt% occurred due to dehydration and side-chain degradation. Subsequently, decomposition of the alginate backbone and polyene residues was observed in the 290–490 °C region. At temperatures above 500 °C, carbonization occurred, accompanied by inorganic residues in the form of Na_2_CO_3_ from the sodium ions in alginate [31,49].

Overall, the primary degradation region of PVA–ALG bio-based polymers (220–290 °C) appeared at significantly lower temperatures compared to PVA–PCD bio-based polymers (250–490 °C) reported in our previous study [30], which can be mainly attributed to the presence of alginate. As shown in the DTG data (Figure S4a), the temperature where thermal weight loss is maximized (T_max_) gradually decreased from 270.3 °C (20 wt% ALG) to 263.8 °C (40 wt% ALG) and 260.9 °C (60 wt% ALG) with increasing the content of ALG. However, at higher temperatures, both the decomposition temperature and residual weight increased with higher alginate content. This result indicates that, at lower temperatures, dehydration and skeletal decomposition of PVA and ALG dominate, whereas at elevated temperatures, decomposition and carbonization of PVA-derived polyenes become the major processes, with a reduced contribution from alginate decomposition.

As illustrated in Figure S4b, the weight loss around 200 °C (~10%) corresponds to the removal of moisture and other volatile compounds. At approximately 300 °C, near T_max_, the residual weight increased with higher alginate content, while at 500 °C, the residue increased from ~20% to ~30%. In comparison with the results of our previous study, crosslinked pure PVA showed only ~8 wt% residue at 500 °C, whereas PVA–ALG bio-based polymers with 20–60 wt% alginate exhibited significantly higher residues of 19–31 wt%. This weight increase originates from the inorganic residues during the thermal degradation of alginate. Overall, these results suggest that the incorporation of alginate substantially enhances the thermal stability of PVA–ALG bio-based polymers [48].

The effect of GLU content on the thermal stability of the PVA–ALG bio-based polymer was investigated (Figure 2c and Figure S4c,d). Figure 2c presents the TGA results of the PVA–ALG bio-based polymer with a PVA:ALG ratio of 60:40, where the GLU crosslinker content was adjusted to 10 and 20 wt%. Regardless of the crosslinker content, no significant differences in the thermal weight loss behavior were observed, and similar results were obtained for other PVA–ALG compositions. In other words, the thermal stability did not show a clear difference between 10 wt% and 20 wt% GLU content. However, the maximum degradation temperature (T_max_) was 264 °C, approximately 20 °C higher than that of the pure alginate sample (ALG, Figure S4e, 245 °C), indicating that alginate was effectively crosslinked with PVA. This result suggests that under the current crosslinker content, the crosslinking between PVA and ALG was already sufficient. Moreover, thermal stability was found to be more strongly influenced by the alginate content than by the crosslinker content. Finally, Figure 2d and Figure S4f,g show the TGA results of PVA–ALG bio-based polymers containing 1 and 2 wt% PCD. The addition of PCD caused negligible changes in the thermal weight loss behavior because of its relatively low content compared to PVA–ALG, resulting in minimal effect on the overall thermal stability.

### 3.3. Swelling Test

**Figure 3:**
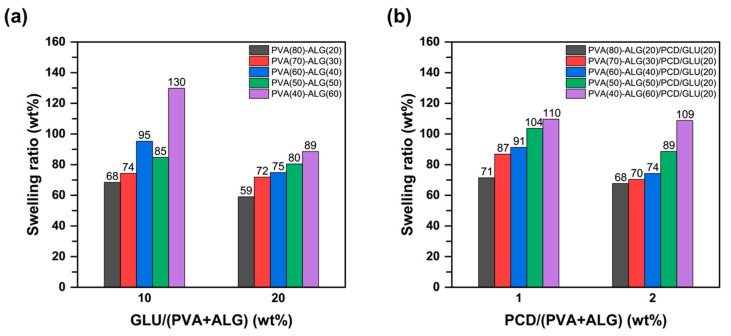
Swelling ratio of (**a**) PVA–ALG (**b**) PVA–ALG/PCD bio-based polymers (Cycle 2-PBS).

We evaluated the swelling behavior of PVA–ALG bio-based polymers, and the results are presented in Figure 3. As shown in Figure 3a, the swelling ratio progressively increased with higher ALG content at the same GLU concentration. Moreover, regardless of the GLU content, the increase in ALG consistently led to higher swelling ratios, suggesting that sufficient crosslinking occurred within the range of 10–20 wt% of the crosslinker. Notably, the swelling ratio of PVA–ALG bio-based polymers remained significantly higher compared with that of PVA–PCD bio-based polymers (12–52% swelling ratio at 10–20 wt% GLU content) [30]. This phenomenon can be attributed to the presence of carboxyl and ether groups in the intrinsic structure of ALG, which can form hydrogen bonds with water molecules, even though some hydroxyl groups were consumed during crosslinking. Therefore, the hygroscopicity and hydrophilicity of ALG played a crucial role in enhancing the swelling capacity of PVA–ALG bio-based polymers.

Figure 3b illustrates the swelling behavior of PVA–ALG/PCD bio-based polymers prepared with a fixed concentration of 20 wt% GLU and added with 1 or 2 wt% of PCD. In all compositions, the swelling performance of PVA–ALG/PCD bio-based polymers varied considerably depending on the PVA:ALG ratio. In particular, an increase in PCD content from 1 to 2 wt% led to a further reduction in swelling ratios, indicating an enhancement of hydrophobic characteristics.

These results suggest that in order to consider both the swelling behavior and antimicrobial properties of the bio-based polymer, the PVA:ALG ratio and the content of PCD polyol should be appropriately controlled. For instance, when the ALG:PVA content was 90:10 wt% under a 10 wt% crosslinker, the swelling degree remained approximately 90% [31]. This result indicates that, due to the low PVA content, swelling is predominantly governed by alginate, and crosslinking did not affect the swelling ratio.

Previous studies have reported that in physically mixed PVA–ALG bio-based polymers, where the hydrophilicity of the hydroxyl and carboxyl groups in both PVA and ALG are retained, the swelling ratio increased with higher ALG content [33,54,55]. In our case, the swelling ratio similarly increased with increasing alginate content, although the degree of increase was relatively modest. This result suggests that chemical crosslinking, which forms strong covalent bonds, has a significant impact on the swelling behavior of bio-based polymers.

In the fabrication of bio-based polymers, simple physical mixing significantly enhances the swelling property but might limit mechanical properties, whereas chemical crosslinking slightly reduces swelling while improving mechanical strength. Therefore, the choice of an appropriate fabrication method of bio-based polymers should be carefully considered depending on the application of the blended bio-based polymer. Moreover, by adjusting the PVA-to-ALG ratio and the concentration of PCD, the physical properties and swelling behavior of PVA–ALG bio-based polymers can be effectively controlled.

### 3.4. Antibacterial Test

**Figure 4:**
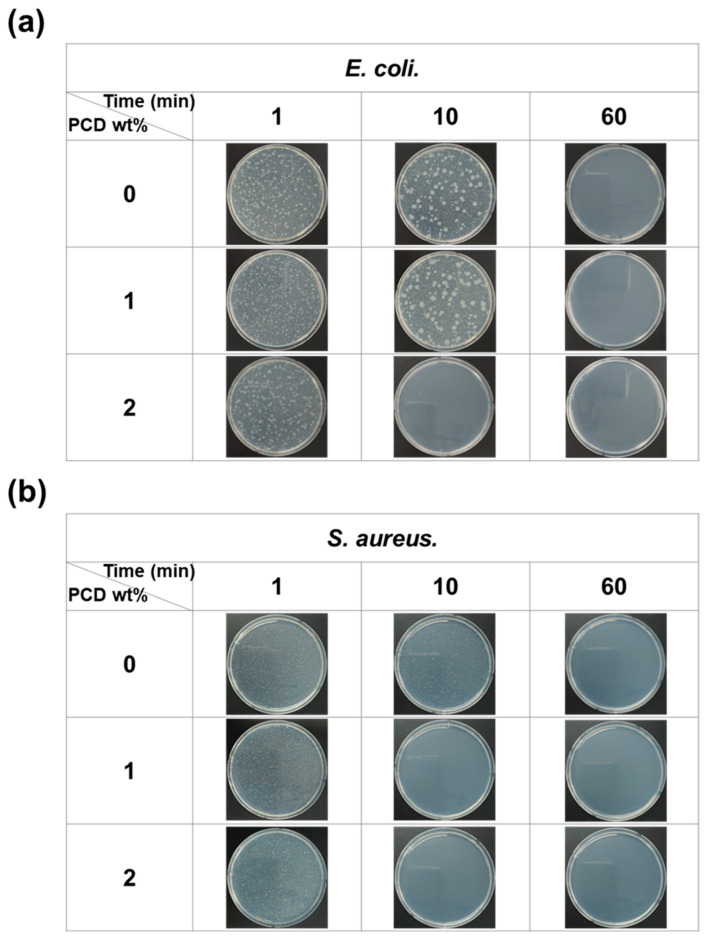
Images of (**a**) *E. coli* and (**b**) *S. aureus.* reduction in PVA(80)–ALG(20)/PCD/GLU(20) bio-based polymers on time.

**Figure 5:**
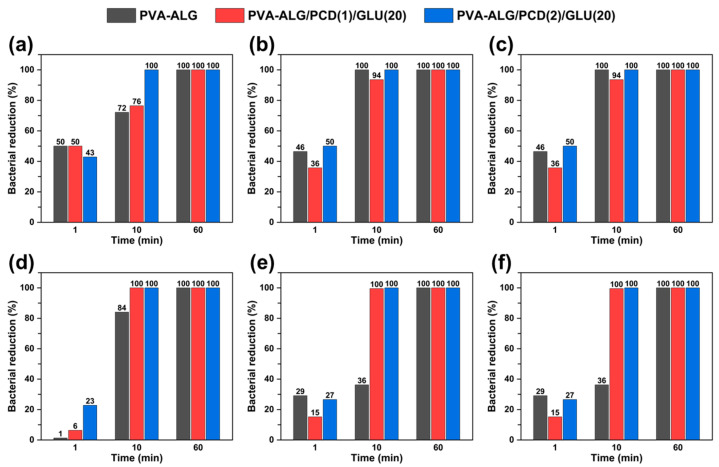
Time-dependent bacterial reduction in PVA–ALG/PCD bio-based polymers (**a**–**c**): *E. coli* and (**d**–**f**): *S. aureus.* (Gray, red, and blue correspond to PVA–ALG, PVA–ALG/PCD(1)/GLU(20), and PVA–ALG/PCD(2)/GLU(20), with PVA:ALG ratios of (**a**,**d**): 80:20, (**b**,**e**): 60:40, and (**c**,**f**): 40:60, respectively).

To evaluate the antibacterial activity of PCD containing PVA–ALG bio-based polymers, two representative pathogens (*E. coli* and *S. aureus*) were tested at exposure times of 1, 10, and 60 min. The images are shown in Figure 4 (PVA:ALG = 80:20) and Figures S6–S9. Also, the bacterial reduction graphs on time are presented in Figure 5.

As shown in Figure 5, both bacterial species reached 100% mortality after 60 min of exposure, indicating that 60 min is sufficient to demonstrate the antibacterial effect of the PVA–ALG bio-based polymers. For the PVA:ALG ratio of 80:20 (Figure 5a), the mortality rate of *E. coli* after 1 min was approximately 43–50%, with no significant differences with PCD contents. In contrast, for *S. aureus* (Figure 5d), a distinct effect of PCD was observed, and the sample containing 2 wt% PCD showed the highest mortality of 23%. After 10 min of exposure, the bio-based polymer containing 2 wt% PCD exhibited complete inhibition of *E. coli*, confirming its strong antibacterial activity.

In the case of the PVA:ALG ratio of 60:40 (Figure 5b,e), the antibacterial effect against *E. coli* was not as pronounced as in the 80:20 composition. However, for *S. aureus*, both PCD-containing bio-based polymers achieved 100% mortality after 10 min, whereas the 0 wt% sample exhibited only 36% mortality. This result suggests that PCD exerts a relatively stronger antibacterial effect against *S. aureus* than against *E. coli*.

When the alginate content was further increased to 60 wt% (Figure 5c,f), both pathogens were completely eradicated within 10 min of exposure. Even at the shortest exposure time (1 min), mortality rates of 50–60% were observed, which were higher than those of the other compositions. This result demonstrates the intrinsic antibacterial activity of alginate at high alginate contents [56,57].

In summary, at low ALG content, the antibacterial effect of PCD is predominant, while at 60 wt% ALG, both the phenolic group-mediated antibacterial activity of PCD and the inherent antibacterial property of ALG act synergistically.

## 4. Conclusions

In this study, bio-based polymers prepared from petroleum-derived biodegradable PVA combined with alginate (ALG) derived from marine biomass and cardanol-derived polyols (PCD) from cashew nut shells were fabricated. The properties of the novel bio-based polymers were systematically characterized using FT-IR, TGA, and XRD and their functional properties were evaluated by the swelling and antibacterial test.

Under variations in the PVA:ALG weight ratio (20–60 wt%), the degree of crosslinking in bio-based polymers occurred sufficiently. As the ALG content increased, the residual mass after decomposition became higher, indicating improved thermal stability, while the swelling ratio gradually increased. These results suggest that the intrinsic pyranose ring structure of ALG restricts extensive crosslinking with PVA, and that the thermal stability and swelling behavior of the bio-based polymer strongly depend on the ALG content.

The incorporation of PCD into PVA–ALG bio-based polymers demonstrated distinct antibacterial activity against *Escherichia coli* and *Staphylococcus aureus*. Specifically, when the incubation time exceeded 10 min, the survival rate of both pathogens decreased by 75–100%.

This work highlights the potential of PVA–ALG–PCD bio-based polymers prepared as eco-friendly and biocompatible materials. The applications would include medical fields (wound dressings and sutures), food and pharmaceutical packaging, filtration membranes for wastewater treatment, antifouling coatings for marine and industrial equipment, and consumer hygiene products.

## Data Availability

Data are contained within the article and Supplementary Materials.

## Supplementary Materials

The following supporting information can be downloaded at https://www.mdpi.com/article/10.3390/polym17202792/s1, Figure S1: FT-IR spectra of PVA, ALG, and PCD raw materials; Figure S2: FT-IR spectra of (a) PVA–ALG/PCD(1) (b) PVA–ALG/PCD(2) bio-based polymers; Figure S3: (a) XRD patterns and (b) crystallinity index of PVA(60)–ALG(40)/PCD/GLU(20) bio-based polymers; Figure S4: (a) DTG curves, (b) residual weight for each temperature for PVA–ALG/GLU(20) bio-based polymers, TGA curves for (c) PVA(80)–ALG(20)/GLU, (d) PVA(40)–ALG(60)/GLU bio-based polymers, (e) DTG curves for PVA(60)–ALG(40)/GLU bio-based polymers and TGA curves for (f) PVA(80)–ALG(20)/PCD/GLU(20), (g) PVA(60)–ALG(40)/PCD/GLU(20) bio-based polymers; Figure S5: Swelling ratio of (a) PVA–ALG (b) PVA–ALG/PCD bio-based polymers (Cycle 1-CaCl_2_ solution); Figure S6: Images of *E. coli* reduction in PVA(60)–ALG(40)/PCD/GLU(20) bio-based polymers on time; Figure S7: Images of *E. coli* reduction in PVA(40)–ALG(60)/PCD/GLU(20) bio-based polymers on time; Figure S8: Images of *S. aureus* reduction in PVA(60)–ALG(40)/PCD/GLU(20) bio-based polymers on time; Figure S9: Images of *S. aureus* reduction in PVA(40)–ALG(60)/PCD/GLU(20) bio-based polymers on time.; Table S1: List of Samples Used in This Study; Table S2: Assignment of functional groups associated with major vibration bands in PVA, ALG, PCD; Table S3: Assignment of functional groups associated with major vibration bands in PVA–ALG bio-based polymer and PVA–ALG/PCD bio-based polymer. References [58,59,60] are cited in the supplementary materials.

## References

[1] Geyer R., Jambeck J.R., Law K.L. (2017). Production, Use, and Fate of All Plastics Ever Made. Sci. Adv..

[2] Costa J.P., Mouneyrac C., Costa M., Duarte A.C., Rocha-Santos T. (2020). The Role of Legislation, Regulatory Initiatives and Guidelines on the Control of Plastic Pollution. Front. Environ. Sci..

[3] Pilapitiya P.N., Ratnayake A.S. (2024). The World of Plastic Waste: A Review. Clean. Mater..

[4] Bin H., Wang S., Yan J., Zhang H., Qiu L., Liu W., Guo Y., Shen J., Bin C., Shi C. (2024). Review of Waste Plastics Treatment and Utilization: Efficient Conversion and High Value Utilization. Process Saf. Environ. Prot..

[5] Kim M.S., Chang H., Zheng L., Yan Q., Pfleger B.F., Klier J., Nelson K., Majumder E.L.-W., Huber G.W. (2023). A Review of Biodegradable Plastics: Chemistry, Applications, Properties, and Future Research Needs. Chem. Rev..

[6] Stoica M., Bichescu C.I., Crețu C.-M., Dragomir M., Ivan A.S., Podaru G.M., Stoica D., Stuparu-Crețu M. (2024). Review of Bio-Based Biodegradable Polymers: Smart Solutions for Sustainable Food Packaging. Foods.

[7] Pawar S.N., Edgar K.J. (2012). Alginate Derivatization: A Review of Chemistry, Properties and Applications. Biomaterials.

[8] Patel M.A., AbouGhaly M.H.H., Schryer-Praga J.V., Chadwick K. (2017). The Effect of Ionotripic Gelation Residence Time on Alginate Cross-Linking and Properties. Carbohydr. Polym..

[9] Raus R.A., Wan Nawawi W.M.F., Nasaruddin R.R. (2021). Alginate and Alginate Composites for Biomedical Applications. Asian J. Pharm. Sci..

[10] Puscaselu R.G., Lobiuc A., Dimian M., Covasa M. (2020). Alginate: From Food Industry to Biomedical Applications and Management of Metabolic Disorders. Polymers.

[11] Thong C.C., Teo D.C.L., Ng C.K. (2016). Application of Polyvinyl Alcohol in Cement-Based Composite Materials: A Review of Its Engineering Properties and Micro-Structure Behavior. Constr. Build. Mater..

[12] Mansur H.S., Orefice R.L., Mansur A.A.P. (2004). Characterization of Poly(vinyl Alcohol)/Poly(ethylene Glycol) Hydrogels and PVA-Derived Hybrids by Small-Angle X-Ray Scattering and FTIR Spectroscopy. Polymer.

[13] Wang X., Yucel T., Lu Q., Hu X., Kaplan D.L. (2010). Silk Nanospheres and Microspheres from Silk/PVA Blend Films for Drug Delivery. Biomaterials.

[14] Khanna P.K., Gokhale R.R., Subbarao V.V.V.S., Singh N., Jun K.W., Das B.K. (2005). Synthesis and Optical Properties of CdS/PVA Nanocomposites. Mater. Chem. Phys..

[15] Zhong Q., Shi G., Sun Q., Mu P., Li J. (2021). Robust PVA-GO-TiO2 Composite Membrane for Efficient Separation Oil-in-Water Emulsions with Stable High Flux. J. Membr. Sci..

[16] Liang X., Zhong H.-J., Ding H., Yu B., Ma X., Liu X., Chong C.-M., He J. (2024). Polyvinyl Alcohol (PVA)-Based Hydrogels: Recent Progress in Fabrication, Properties, and Multifunctional Applications. Polymers.

[17] Wang M., Bai J., Shao K., Tang W., Zhao X., Lin D., Huang S., Chen C., Ding Z., Ye J. (2021). Poly(Vinyl Alcohol) Hydrogels: The Old and New Functional Materials. Int. J. Polym. Sci..

[18] Kim J.O., Park J.K., Kim J.H., Jin S.G., Yong C.S., Li D.X., Choi J.Y., Woo J.S., Yoo B.K., Lyoo W.S. (2008). Development of Polyvinyl Alcohol–Sodium Alginate Gel-Matrix-Based Wound Dressing System Containing Nitrofurazone. Int. J. Pharm..

[19] Tarun K., Gobi N. (2012). Calcium Alginate/PVA Blended Nanofibre Matrix for Wound Dressing. Indian J. Fibre Text. Res..

[20] Kamel S., Dacrory S., Hesemann P., Bettache N., Ali L.M.A., Postel L., Akl E.M., El-Sakhawy M. (2023). Wound Dressings Based on Sodium Alginate-Polyvinyl Alcohol-Moringa oleifera Extracts. Pharmaceutics.

[21] Motelica L., Ficai D., Oprea O., Ficai A., Trusca R.-D., Andronescu E., Holban A.M. (2021). Biodegradable Alginate Films with ZnO Nanoparticles and Citronella Essential Oil—A Novel Antimicrobial Structure. Pharmaceutics.

[22] Voirin C., Caillol S., Sadavarte N.V., Tawade B.V., Boutevin B., Wadgaonkar P.P. (2014). Functionalization of Cardanol: Towards Biobased Polymers and Additives. Polym. Chem..

[23] Lubi M.C., Thachil E.T. (2000). Cashew Nut Shell Liquid (CNSL)—A Versatile Monomer for Polymer Synthesis. Des. Monomers Polym..

[24] Balachandran V.S., Jadhav S.R., Vemula P.K., John G. (2013). Recent Advances in Cardanol Chemistry in a Nutshell: From a Nut to Nanomaterials. Chem. Soc. Rev..

[25] Kanehashi S., Masuda R., Yokoyama K., Kanamoto T., Nakashima H., Miyakoshi T. (2015). Development of a Cashew Nut Shell Liquid (CNSL)-Based Polymer for Antibacterial Activity. J. Appl. Polym. Sci..

[26] Pan Z., Ye H., Wu D. (2021). Recent Advances on Polymeric Hydrogels as Wound Dressings. APL Bioeng..

[27] Jahan A., Masood S., Zafar F., Shaily, Rizvi S.A., Alam M., Ghosal A., Haq Q.M.R., Nishat N. (2024). Development of Cardanol-Based Antibacterial and Anticorrosive Bio-Polyurea-Epoxy Composite Coating for Mild Steel Surface. Prog. Org. Coat..

[28] Wang T., Zhang F., Zhao R., Wang C. (2020). Polyvinyl Alcohol/Sodium Alginate Hydrogels Incorporated with Silver Nanoclusters via Green Tea Extract for Antibacterial Applications. Des. Monomers Polym..

[29] Rafiq M., Hussain T., Abid S., Nazir A., Masood R. (2018). Development of Sodium Alginate/PVA Antibacterial Nanofibers by the Incorporation of Essential Oils. Mater. Res. Express.

[30] Lee D.H., Song Y.H., Ahn H.J., Lee J., Woo H.C. (2024). Fabrication and Characterization of Biopolymers Using Polyvinyl Alcohol and Cardanol-Based Polyols. Molecules.

[31] Ahn H.J., Kang K.S., Song Y.H., Lee D.H., Kim M.H., Lee J.K., Woo H.C. (2022). Effect of Cardanol Content on the Antibacterial Films Derived from Alginate-PVA Blended Matrix. Clean Technol..

[32] Abral H., Atmajaya A., Mahardika M., Hafizulhaq F. (2020). Effect of ultrasonication duration of polyvinyl alcohol (PVA) gel on characterizations of PVA film. J. Mater. Res. Technol..

[33] Afshar M., Dini G., Vaezifar S., Mehdikhani M., Movahedi B. (2020). Preparation and Characterization of Sodium Alginate/Polyvinyl Alcohol Hydrogel Containing Drug-Loaded Chitosan Nanoparticles as a Drug Delivery System. J. Drug Delivery Sci. Technol..

[34] Xu Y., Xu Y., Sun C., Zou L., He J.W. (2020). The preparation and Characterization of Plasticized PVA Fibres by A novel Glycerol/Pseudo Ionic Liquids System with Melt Spinning Method. Eur. Polym. J..

[35] Yang W., Qi G., Kenny J.M., Puglia D., Ma P. (2020). Effect of Cellulose Nanocrystals and Lignin Nanoparticles on Mechanical, Antioxidant and Water Vapour Barrier Properties of Glutaraldehyde Crosslinked PVA Films. Polymers.

[36] Wang H., Zhang R., Zhang H., Jiang S., Liu H., Sun M., Jiang S. (2015). Kinetics and Functional Effectiveness of Nisin Loaded Antimicrobial Packaging Film Based on Chitosan/Poly(vinyl alcohol). Carbohydr. Polym..

[37] Raptopoulos G., Choinopoulos I., Kontoes-Georgoudakis F., Paraskevopoulou P. (2022). Polylactide-Grafted Metal-Alginate Aerogels. Polymers.

[38] Cardenas-Jiron G., Leal D., Matsuhiro B., Osorio-Roman I. (2011). Vibrational Spectroscopy and Density Functional Theory Calculations of Poly-D-Mannuronate and Heteropolymeric Fractions from Sodium Alginate. J. Raman Spectrosc..

[39] Sakugawa K., Ikeda A., Takemura A., Ono H. (2004). Simplified Method for Estimation of Composition of Alginates by FTIR. J. Appl. Polym. Sci..

[40] Xiao Q., Gu X., Tan S. (2014). Drying Process of Sodium Alginate Films Studied by Two-dimensional Correlation ATR-FTIR Spectroscopy. Food Chem..

[41] Liu Z., Chen J., Knothe G., Nie X., Jiang J. (2016). Synthesis of Epoxidized Cardanol and Its Antioxidative Properties for Vegetable Oils and Biodiesel. ACS Sustain. Chem. Eng..

[42] Li S., Yang X., Huang K., Li M., Xia J. (2014). Design, Preparation and Properties of Novel Renewable UV-Curable Copolymers Based on Cardanol and Dimer Fatty Acids. Prog. Org. Coat..

[43] Shukla P., Srivastava D. (2014). Reaction Kinetics of Esterification of Phenol-Cardanol Based Epoxidized Novolac Resins and Methacrylic Acid. Int. J. Plast. Technol..

[44] Hu Y., Shang Q., Bo C., Jia P., Feng G., Zhang F., Liu C., Zhou Y. (2019). Synthesis and Properties of UV-Curable Polyfunctional Polyurethane Acrylate Resins from Cardanol. ACS Omega.

[45] Choi D., Khan M.H., Jung J. (2019). Crosslinking of PVA/Alginate Carriers by Glutaraldehyde with Improved Mechanical Strength and Enhanced Inhibition of Deammonification Sludge. Int. Biodeterior. Biodegrad..

[46] Li X., Shu M., Li H., Gao X., Long S., Hu T., Wu C. (2018). Strong, Tough and Mechanically Self-Recoverable Poly(Vinyl Alcohol)/Alginate Dual-Physical Double-Network Hydrogels with Large Cross-Link Density Contrast. RSC Adv..

[47] Rudra R., Kumar V., Kundu P.P. (2015). Acid Catalysed Cross-Linking of Poly(Vinyl Alcohol) (PVA) by Glutaraldehyde: Effect of Crosslink Density on the Characteristics of PVA Membranes Used in Single Chambered Microbial Fuel Cells. RSC Adv..

[48] Meng W., Zhang X., Zhang Y., Zhang X. (2023). Poly(Vinyl Alcohol)/Sodium Alginate Polymer Membranes as Eco-Friendly and Biodegradable Coatings for Slow Release Fertilizers. J. Sci. Food Agric..

[49] Freire T.F., Quinaz T., Fertuzinhos A., Quyền N.T., de Moura M.F.S.M., Martins M., Zille A., Dourado N. (2021). Thermal, Mechanical and Chemical Analysis of Poly(Vinyl Alcohol) Multifilament and Braided Yarns. Polymers.

[50] Aydın A.A., Ilberg V. (2016). Effect of Different Polyol-Based Plasticizers on Thermal Properties of Polyvinyl Alcohol: Starch Blends. Carbohydr. Polym..

[51] Yang J.M., Yang J.H., Tsou S.C., Ding C.H., Hsu C.C. (2016). Cell Proliferation on PVA/Sodium Alginate and PVA/Poly(γ-Glutamic Acid) Electrospun Fiber. Mater. Sci. Eng. C.

[52] Siddaramaiah, Swamy T.M.M., Ramaraj B., Lee J.H. (2008). Sodium Alginate and Its Blends with Starch: Thermal and Morphological Properties. J. Appl. Polym. Sci..

[53] Rodrigues F.H.A., Souza J.R.R., França F.C.F., Ricardo N.M.P.S., Feitosa J.P.A. (2006). Thermal Oligomerisation of Cardanol. e-Polymers.

[54] Kamoun E.A., Kenawy E.R.S., Tamer T.M., El-Meligy M.A., Eldin M.S.M. (2015). Poly(Vinyl Alcohol)-Alginate Physically Crosslinked Hydrogel Membranes for Wound Dressing Applications: Characterization and Bio-Evaluation. Arabian J. Chem..

[55] Shivakumara L.R., Demappa T. (2019). Synthesis and Swelling Behavior of Sodium Alginate/Poly(Vinyl Alcohol) Hydrogels. Turk. J. Pharm. Sci..

[56] Salem D.M.S.A., Sallam M.A.E., Youssef T.N.M.A. (2019). Synthesis of Compounds Having Antimicrobial Activity from Alginate. Bioorg. Chem..

[57] Asadpoor M., Ithakisiou G.-N., van Putten J.P.M., Pieters R.J., Folkerts G., Braber S. (2021). Antimicrobial Activities of Alginate and Chitosan Oligosaccharides Against Staphylococcus aureus and Group B Streptococcus. Front. Microbiol..

[58] Yumin A., Liguo D., Yi Y., Yongna J. (2022). Mechanical Properties of an Interpenetrating Network Poly(vinyl alcohol)/Alginate Hydrogel with Hierarchical Fibrous Structures. RSC Adv..

[59] Mansur H.S., Sadahira C.M., Souza A.N., Mansur A.A.P. (2008). Ftir Spectroscopy Characterization of Poly (Vinyl Alcohol) Hydrogel with Different Hydrolysis Degree and Chemically Crosslinked with Glutaraldehyde. Mater. Sci. Eng. C.

[60] Pandit A.H., Mazumdar N., Imtiyaz K., Rizvi M.M.A., Ahmad S. (2019). Periodate-Modified Gum Arabic Cross-Linked PVA Hydrogels: A Promising Approach toward Photoprotection and Sustained Delivery of Folic Acid. ACS Omega.

