# Supplementary material for: Biodegradable PVA–Alginate Bio-Based Polymers Incorporating Cardanol-Based Polyols for Antibacterial Applications
Source: Polymers (Basel). 2025 Oct 18;17(20):2792. doi: 10.3390/polym17202792 (PMC12567143; doi:10.3390/polym17202792)
Supplement: Supplementary file 1 [file polymers-17-02792-s001.zip › polymers-3880921-supplementary.pdf]

Supplementary files

Biodegradable PVA–Alginate Bio-Based Polymers Incorporating Cardanol-Based Polyols for Antibacterial Applications

Da Hae Lee, Hee Ju Ahn, Jaekyoung Lee * and Hee Chul Woo *

Department of Chemical Engineering, Pukyong National University, 45 Yongso-ro, Nam-gu, Busan 48513, Republic of Korea; ekgo2364@pukyong.ac.kr (D.H.L.)

* Correspondence: leejk46@pknu.ac.kr (J.L.); woohc@pknu.ac.kr (H.C.W.); Tel.: +82-51-629-6433 (J.L.)

Table S1. List of bio-based polymers.

No.	PVA( )–ALG( )/PCD( )/GLU( )	PVA : ALG (w/w)	PCD/(PVA+ALG) (wt%)	GLU/(PVA+ALG) (wt%)
1	PVA(80)–ALG(20)/GLU(10)	8:2	0	10
2	PVA(80)–ALG(20)/PCD(1)/GLU(10)		1	
3	PVA(80)–ALG(20)/PCD(2)/GLU(10)		2	
4	PVA(70)–ALG(30)/GLU(10)	7:3	0	
5	PVA(70)–ALG(30)/PCD(1)/GLU(10)		1	
6	PVA(70)–ALG(30)/PCD(2)/GLU(10)		2	
7	PVA(60)–ALG(40)/GLU(10)	6:4	0	
8	PVA(60)–ALG(40)/PCD(1)/GLU(10)		1	
9	PVA(60)–ALG(40)/PCD(2)/GLU(10)		2	
10	PVA(50)–ALG(50)/GLU(10)	5:5	0	
11	PVA(50)–ALG(50)/PCD(1)/GLU(10)		1	
12	PVA(50)–ALG(50)/PCD(2)/GLU(10)		2	
13	PVA(40)–ALG(60)/GLU(10)	4:6	0	
14	PVA(40)–ALG(60)/PCD(1)/GLU(10)		1	
15	PVA(40)–ALG(60)/PCD(2)/GLU(10)		2	
16	PVA(80)–ALG(20)/GLU(20)	8:2	0	20
17	PVA(80)–ALG(20)/PCD(1)/GLU(20)		1	
18	PVA(80)–ALG(20)/PCD(2)/GLU(20)		2	
19	PVA(70)–ALG(30)/GLU(20)	7:3	0	
20	PVA(70)–ALG(30)/PCD(1)/GLU(20)		1	
21	PVA(70)–ALG(30)/PCD(2)/GLU(20)		2	
22	PVA(60)–ALG(40)/GLU(20)	6:4	0	
23	PVA(60)–ALG(40)/PCD(1)/GLU(20)		1	
24	PVA(60)–ALG(40)/PCD(2)/GLU(20)		2	
25	PVA(50)–ALG(50)/GLU(20)	5:5	0	
26	PVA(50)–ALG(50)/PCD(1)/GLU(20)		1	
27	PVA(50)–ALG(50)/PCD(2)/GLU(20)		2	
28	PVA(40)–ALG(60)/GLU(20)	4:6	0	
29	PVA(40)–ALG(60)/PCD(1)/GLU(20)		1	
30	PVA(40)–ALG(60)/PCD(2)/GLU(20)		2	

Table S2. Assignment of functional groups associated with major vibration bands in PVA, ALG, PCD.

Functional group		Wavenumber (cm^{-1})	Ref.
O-H stretching at hydrogen bond system		3200–3500	[32,34]
C-H stretching from aliphatic chain		2925, 2853	[35,36]
C=C stretching of aromatic ring		1589, 1487	[41,42]
O-C-O asymmetric Stretching (carboxyl anions)		1590, 1415	[37]
In-plane bending of C-H in alkyl chain		1456	[35]
C-O stretching between phenolic hydroxyl and aromatic ring		1263	[34]
Pyranose ring of ALG	C-C-H and O-C-H deformation	1313	[37–40]
	C-O stretching	1123	
	C-O and C-C stretching	1080	
C-H bending vibration in aromatic ring		911, 884	[41,42]
Out-of-plane bending of C-H in aromatic ring		773, 692	[41]

Table S3. Assignment of functional groups associated with major vibration bands in PVA–ALG bio-based polymer and PVA–ALG/PCD bio-based polymer.

Functional group		Wavenumber (cm^{-1})	Ref.
O-H stretching at hydrogen bonding		3259–3280	[32,34,58]
C–H stretching vibrations of crosslinked acetal		2910–2940	[36]
C–H stretching vibration of acetal groups		2860	[45]
C=O stretching (free -CHO of GLU, carboxyl, ester, ketone)		1705–1715	[59,60]
O-C-O asymmetric Stretching (carboxyl anions)		1590, 1415	[37]
Pyranose ring of ALG	C-C-H and O-C-H deformation	1313	[37–40]
	C-O stretching	1123	
	C-O and C-C stretching	1080	
Ether linkages of acetal bridges		991–1007	[45,60]
C-H bending vibration in benzene ring		911, 884	[41,42]
Out-of-plane bending of C-H in aromatic ring		773, 692	[41]

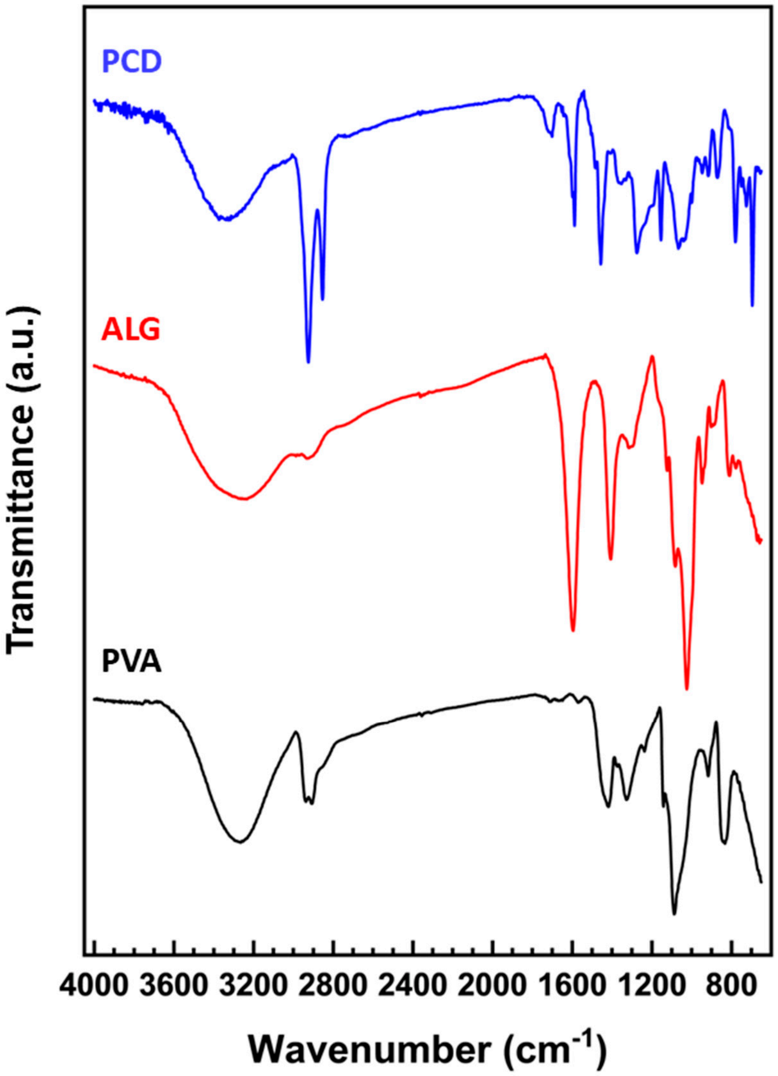

Figure S1. FT-IR spectra of PVA, ALG, and PCD raw materials.

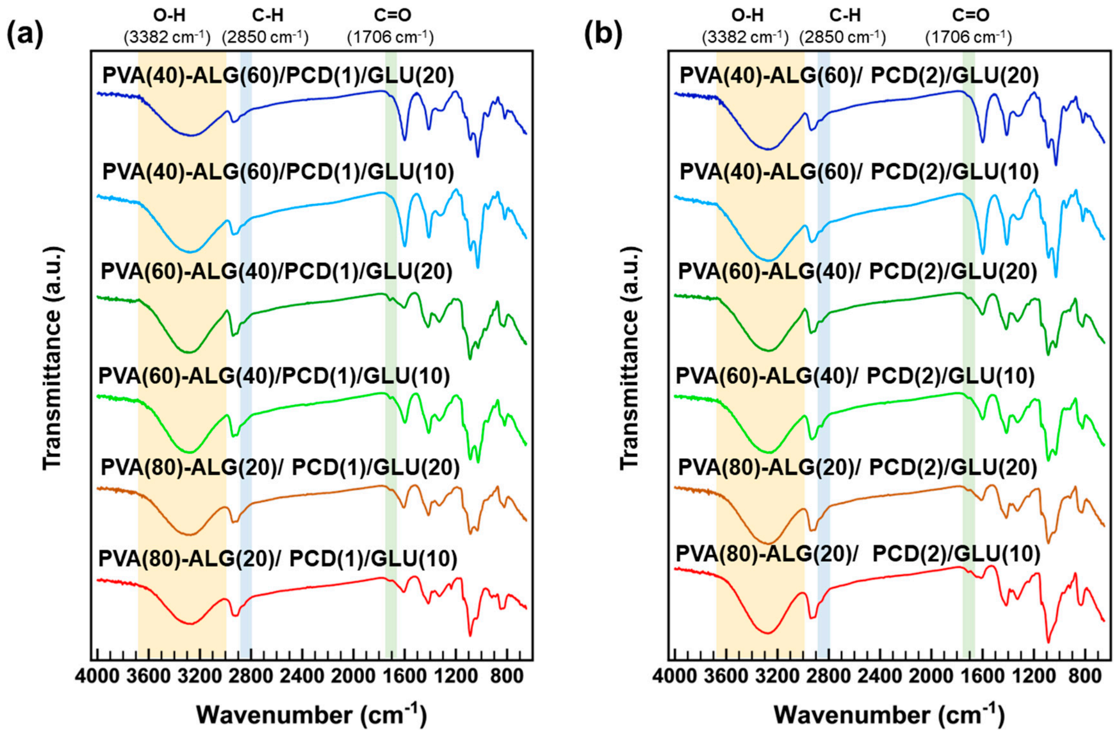

Figure S2. FT-IR spectra of PVA–ALG/PCD bio-based polymers (a) PCD 1 wt%, (b) PCD 2 wt%.

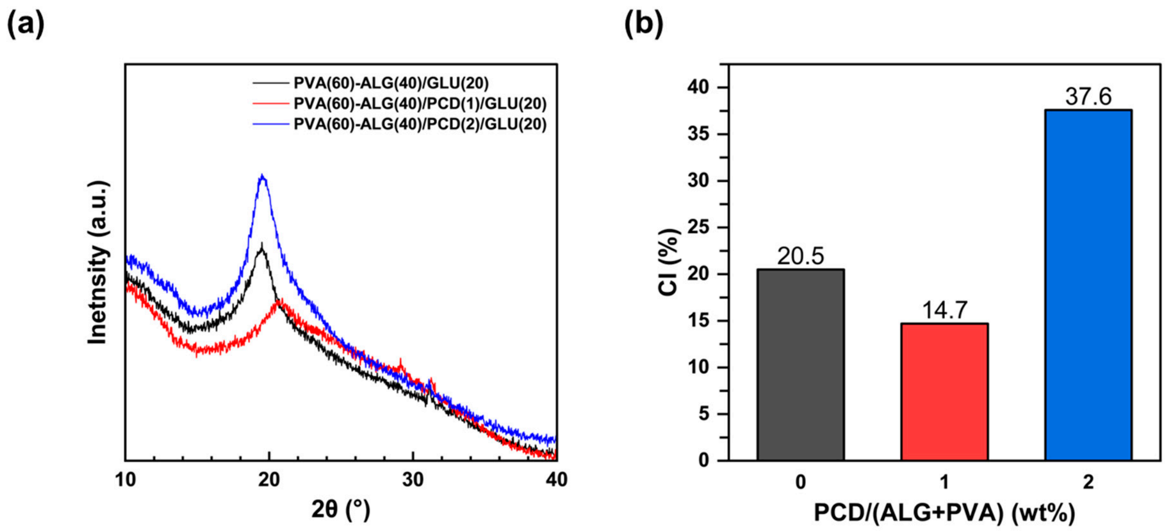

Figure S3. (a) XRD patterns and (b) crystallinity index of PVA(60)–ALG(40)/PCD/GLU(20) bio-based polymers calculated by XRD results.

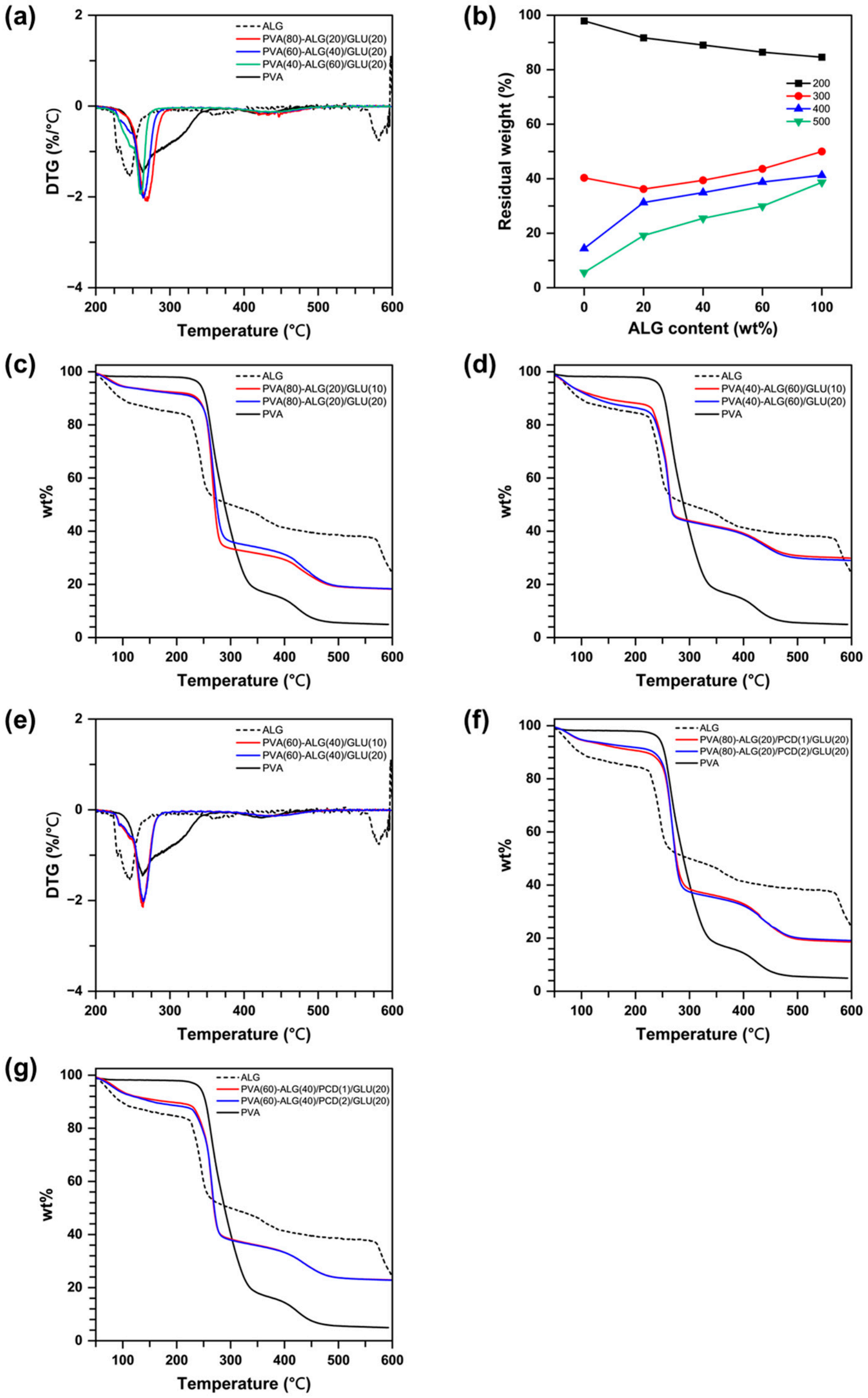

Figure S4. (a) DTG curves, (b) residual weight for each temperature for PVA–ALG/GLU(20) bio-based polymers, TGA curves for (c) PVA(80)–ALG(20)/GLU, (d) PVA(40)–ALG(60)/GLU bio-based polymers, (e) DTG curves for PVA(60)–ALG(40)/GLU bio-based polymers and TGA curves for (f) PVA(80)–

ALG(20)/PCD/GLU(20), (g) PVA(60)–ALG(40)/PCD/GLU(20) bio-based polymers.

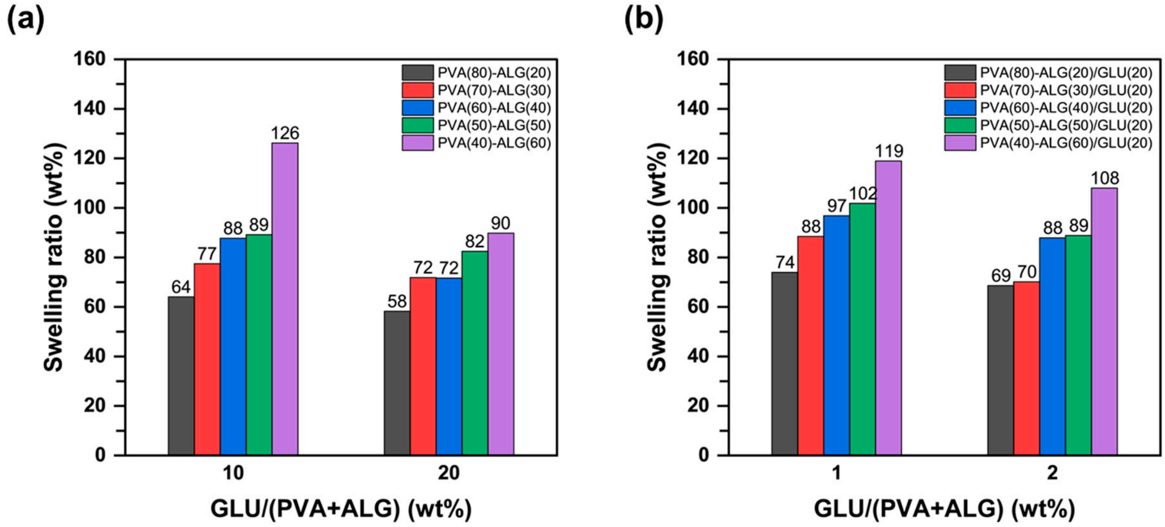

Figure S5. Swelling ratio of (a) PVA–ALG (b) PVA–ALG/PCD bio-based polymers (Cycle 1-$CaCl_2$ solution).

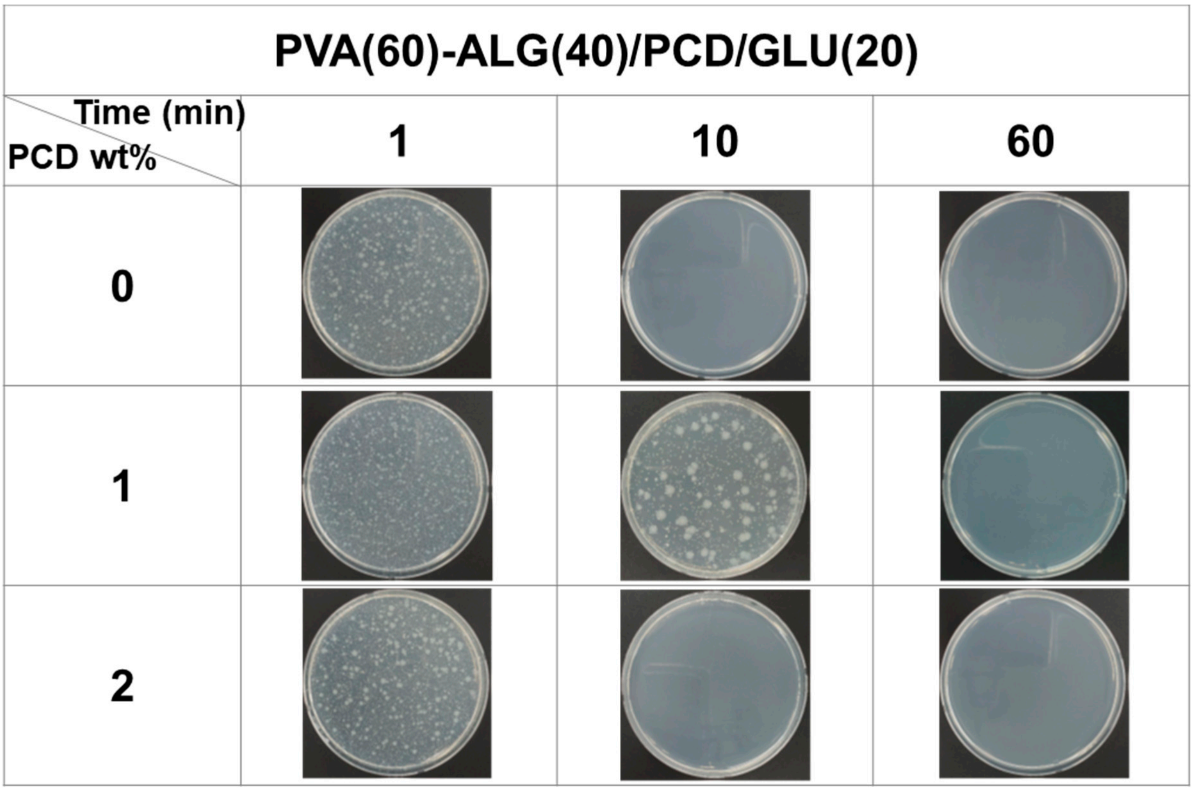

Figure S6. Image of *E. coli* reduction in PVA(60)–ALG(40)/PCD/GLU(20) bio-based polymers on time.

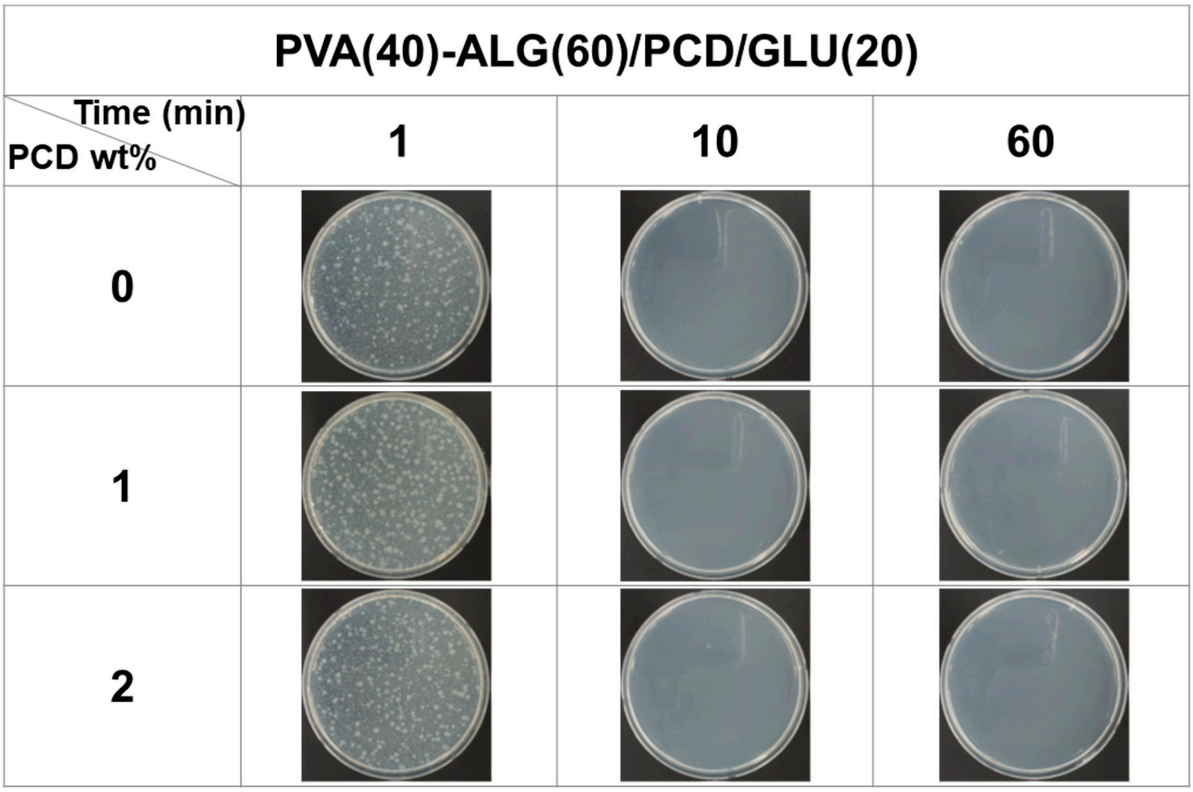

Figure S7. Image of *E. coli* reduction in PVA(40)–ALG(60)/PCD/GLU(20) bio-based polymers on time.

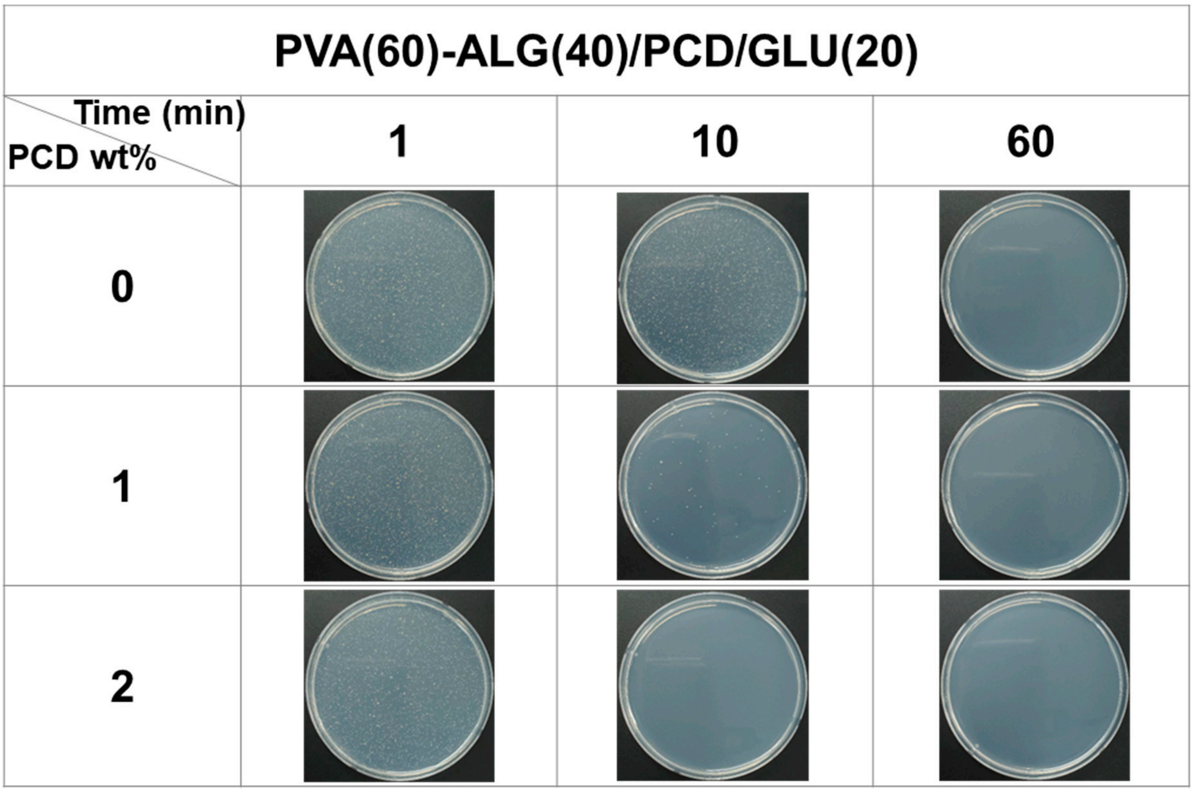

Figure S8. Image of *S. aureus* reduction in PVA(60)–ALG(40)/PCD/GLU(20) bio-based polymers on time.

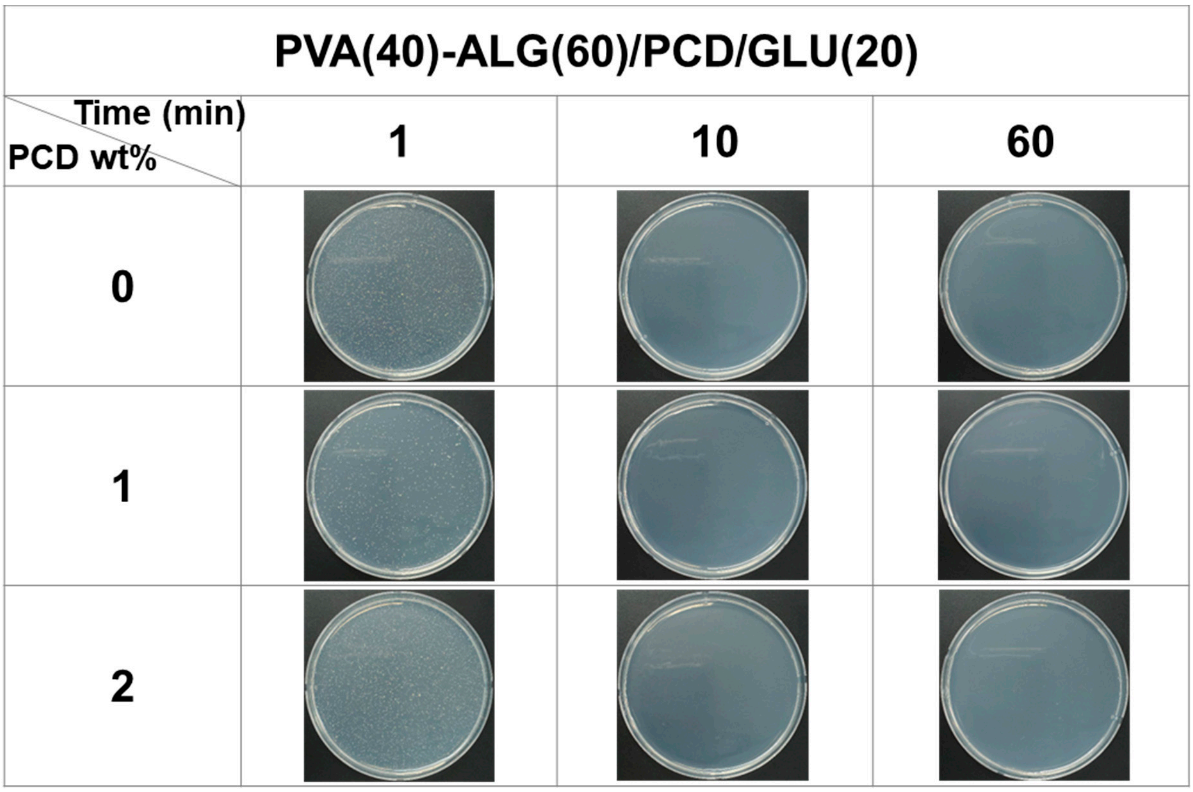

Figure S9. Image of *S. aureus* reduction in PVA(40)–ALG(60)/PCD/GLU(20) bio-based polymers on time.
